# Hypoxia-Inducible Factor-1 in Physiological and Pathophysiological Angiogenesis: Applications and Therapies

**DOI:** 10.1155/2015/549412

**Published:** 2015-06-04

**Authors:** Agnieszka Zimna, Maciej Kurpisz

**Affiliations:** Institute of Human Genetics, Polish Academy of Sciences, Strzeszynska 32, 60-479 Poznań, Poland

## Abstract

The cardiovascular system ensures the delivery of oxygen and nutrients to all cells, tissues, and organs. Under extended exposure to reduced oxygen levels, cells are able to survive through the transcriptional activation of a series of genes that participate in angiogenesis, glucose metabolism, and cell proliferation. The oxygen-sensitive transcriptional activator HIF-1 (hypoxia-inducible factor-1) is a key transcriptional mediator of the response to hypoxic conditions. The HIF-1 pathway was found to be a master regulator of angiogenesis. Whether the process is physiological or pathological, HIF-1 seems to participate in vasculature formation by synergistic correlations with other proangiogenic factors such as VEGF (vascular endothelial growth factor), PlGF (placental growth factor), or angiopoietins. Considering the important contributions of HIF-1 in angiogenesis and vasculogenesis, it should be considered a promising target for treating ischaemic diseases or cancer. In this review, we discuss the roles of HIF-1 in both physiological/pathophysiological angiogenesis and potential strategies for clinical therapy.

## 1. Angiogenesis

The circulatory system is the first biological system that is established during mammalian development [1]. Vessel formation occurs via only two basic mechanisms: vasculogenesis and angiogenesis [2]. During embryonic development, the primary vascular plexus is formed by vasculogenesis. This phenomenon involves* de novo* blood vessel formation from precursor cells called angioblasts (precursors of endothelial cells), whereas angiogenesis is the process by which blood vessels are formed from preexisting vessels. This process involves the remodelling of blood vessels into the large and small vessels that are typical for networks containing arteries, capillaries, and veins [3, 4]. Angiogenesis occurs in adult organisms and in embryos during development [3].

Angiogenesis consists of several basic steps. Briefly, biological signals such as hypoxia, ischaemia, and/or blood vessel damage upregulate the expression of proangiogenic growth factors that activate their receptors [5, 6]. Vascular permeability increases in response to VEGF, thereby allowing the extravasation of plasma proteins that form a primitive scaffold for migrating endothelial cells [7]. Angiopoietin-1 and angiopoietin-2 (Ang-1 and Ang-2) exert antagonistic functions during vessel development. Ang-1, which is a known natural inhibitor of vascular permeability, protects against plasma leakage, whereas Ang-2 is involved in vessel destabilisation via the detachment of smooth muscle cells and the promotion of permeabilisation [8, 9]. Subsequently, matrix metalloproteinases (MMPs) enhance angiogenesis through the degradation of matrix components [10]. Proliferating endothelial cells migrate to distant sites and then assemble as a solid cord that subsequently forms a lumen [11]. Integrins *αβ* promote endothelial cell adhesion and migration, whereas VE-cadherin increases cell survival and promotes endothelial cell adhesion [12–14]. Once the vessels are formed, pericytes and smooth muscle cells surround the newly created capillaries to stabilise the walls and to prevent leakage. Other factors such as Ang-1, PDGF-BB (platelet-derived growth factor BB), and PDGFR (platelet-derived growth factor receptor) participate in the maturation of blood vessels [8, 15]. Further expansion of the lumen diameter is called arteriogenesis [11].

Angiogenesis occurs in physiological states such as embryonic development, wound healing, or vessel penetration into avascular regions and in pathological states such as solid tumours formation, eye diseases, or chronic inflammatory disorders such as rheumatoid arthritis, psoriasis, and periodontitis [16, 17]. Pathophysiological angiogenesis exhibits differences in molecular pathways in comparison to physiological angiogenesis. Mutations in oncogenes and tumour suppressor genes and disruptions in growth factor activity play crucial roles during tumour angiogenesis [17]. The activation of the most prominent proangiogenic factor VEGF might be due to physiological stimuli such as hypoxia or inflammation or due to oncogene activation and tumour suppression function loss [18, 19]. Additionally, physiological angiogenesis such as that which occurs during embryonic development or wound healing seems to be dependent on VEGF signalling, whereas tumour angiogenesis adopts the ability to shift its dependence from VEGF to other proangiogenic pathways, for example, through the recruitment of myeloid cells and the upregulation of alternative vascular growth factors (PlGF and FGF, fibroblast growth factor) [17]. Moreover, tumour vessels are distinct from normal vasculature because they are disorganised and tortuous. Many morphological and functional differences exist between normal and tumour vasculature. For instance, tumour vessels are leakier than normal vessels, and endothelial cells growing within tumours carry genetic abnormalities [20, 21]. Elucidating the molecular mechanism of pathological angiogenesis might lead to the identification of potential therapeutic targets.

Angiogenesis is a multistep process that requires the involvement of many biological signals and stimuli regardless of whether it is a physiological or pathological action. Proangiogenic factors are activated in response to some physical signals. Blood vessel damage, infarction, and blood flow reduction lead to decreases in O_2_ supply [11]. This state is called hypoxia, which is a potent angiogenic trigger that stimulates proangiogenic factor activity.

## 2. Hypoxia

### 2.1. O_2_ Homeostasis

Constant oxygen supply is essential for proper tissue function, development, and homeostasis. Thus, the vasculature network plays a crucial role in delivering oxygen particles (O_2_), nutrients, and other molecules within the entire human body [22]. Significantly, the first physiological system that becomes functional during mammalian embryonic development is the circulatory system. Governing O_2_ homeostasis in tissues by supplying an oxygen concentration adequate for the demand generated by the metabolic outputs of the tissue is essential. Oxygenic balance can be upset by rapid cellular division during embryonic development, by tumour growth, or by vasculature dysfunction due to vessel occlusion or rupture [23]. Notably, normal physiological O_2_ concentrations vary greatly from normal oxygen tension in the air depending on the type of tissue. For instance, arterial blood has a normal pO_2_ of 14%; myocardium, 10%; and skeletal muscle, 5%. In contrast, the natural pO_2_ levels of bone marrow, thymus, and cartilage are at or below 1% [24, 25]. The state when the O_2_ level decreases relative to physiological levels (characteristic for particular tissues) is called hypoxia [1].

### 2.2. Hypoxia-Inducible Factor-1

**Figure 1:**
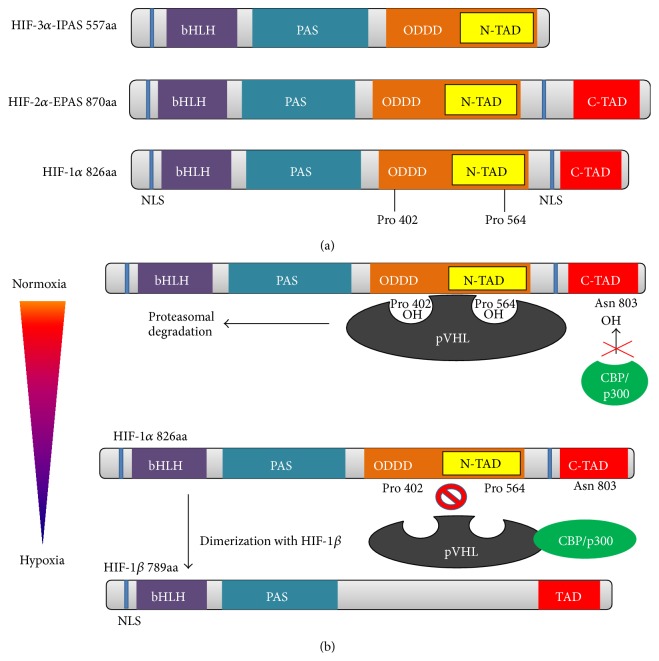
*Schematic representation of HIF-α gene structures and DNA binding*. (a) HIF-1*α* and HIF-2*α* contain the following domains: a nuclear localisation domain (NLS), DNA binding and dimerisation domains (bHLH/PAS), oxygen-dependent degradation domain (ODDD), and cofactor interaction and transcriptional activity domains (N-TAD/C-TAD). HIF-3*α* lacks a C-TAD domain. NLS: nuclear localisation signal; bHLH: basic helix-loop-helix domain; PAS: Per-ARNT-Sim motif; ODDD: oxygen-dependent degradation domain; N-TAD: N-terminal transactivation domain; C-TAD: C-terminal transactivation domain. (b) Dimerisation of HIF-1*α* with HIF-1*β* under hypoxic conditions results in the formation of the HIF-1 transcription factor, which binds to hypoxia response elements (HREs) and activates the transcription of O_2_-dependent genes (according to [134]).

Adaptation to low oxygen tension (hypoxia) in cells and tissues requires the activation of several genes that participate in angiogenesis, cell proliferation/survival, glucose and iron metabolism. In eukaryotic cells, hypoxia-inducible factor-1 (HIF-1) is a primary transcriptional mediator of the hypoxic response and master regulator of O_2_ homeostasis [26]. Hypoxia-inducible factor-1 was first discovered as a transcription factor that regulates erythropoietin (EPO) expression in response to low oxygen levels in the blood [27, 28]. HIF-1 consists of two different subunits, *α* and *β* (also known as aryl hydrocarbon nuclear receptor translocator (ARNT)); both subunits are members of the basic helix-loop-helix Per-Arnt-Sim (bHLH-PAS) transcription factor family [29, 30]. PAS and bHLH motifs are required for heterodimerisation between the HIF-1*α* and HIF-1*β* subunits [31]. Additionally, the bHLH domain of the HIF-1*α*/ARNT dimer is essential for DNA binding on hypoxia response elements (HREs) with the consensus sequence (G/ACGTG) in the promoters or enhancers of target genes [32]. Transcriptional activation and interactions with coactivators (such as CBP/p300) of HIF-1*α* are mediated by two domains, that is, C-TAD and N-TAD, which are on the C-terminus of the protein [33–35]. HIF-1*β* is a constitutively expressed subunit, whereas HIF-1*α* is translated continuously and degraded subsequently through ubiquitination under normoxic conditions [36, 37]. Thus far, three isoforms of HIF-*α* have been discovered (Figure 1(a)), that is, the previously discussed subunit HIF-1*α* and two other subunits, HIF-2*α* (also called endothelial PAS protein (EPAS)) and HIF-3*α* (IPAS). The second isoform is expressed constitutively in the endothelium, lung, and cartilage and shares 48% amino acid sequence identity with HIF-1*α*. The third protein, HIF-3*α*, also called inhibitory PAS (IPAS), is a negative regulator of HIF-1 that dimerises with the HIF-1*α* subunit and prevents its DNA-binding activity. The entire set of HIF-*α* subunits dimerises with ARNT and binds to HREs [38–40].

**Figure 2:**
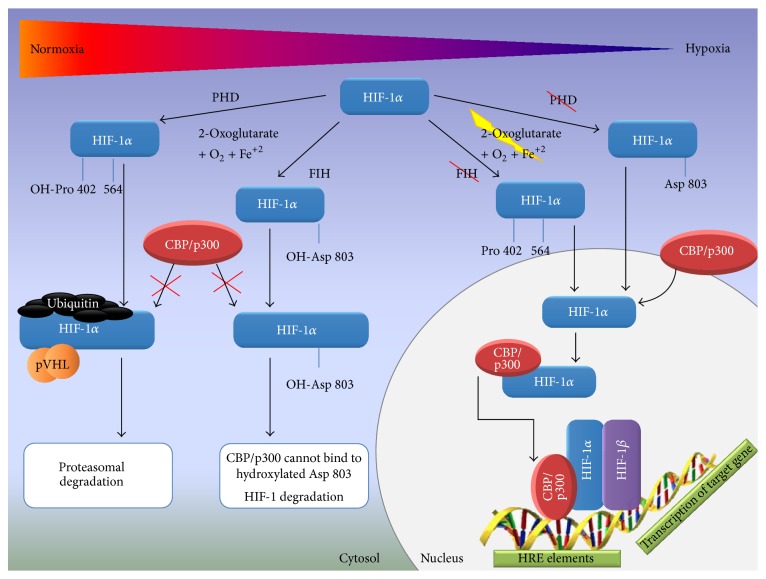
*HIF-1α under normoxic and hypoxic conditions.* In the presence of molecular oxygen, 2-oxoglutarate, and Fe^2+^, HIF-1*α* is hydroxylated on proline 402 and 564 residues located within ODDD (O_2_-dependent degradation domain) by prolyl hydroxylase enzymes (PHDs). Hydroxylation results in the binding of the von Hippel-Lindau (VHL) E3 ligase complex, which ubiquitinates HIF-1*α*, targeting it to proteasomal degradation. The hydroxylation of the asparagine residue prevents CBP/p300 binding to HIF-1*α*. Under hypoxic conditions, substrates and coactivators of hydroxylation such as O_2_, Fe(II), and 2-oxoglutarate become limited, which leads to the attenuation of HIF-1*α* hydroxylation. HIF-1*α* accumulates in the cytosol and is subsequently translocated into the nucleus where it dimerises with the HIF-1*β* subunit. The HIF-1*α*/*β* dimer binds to HREs and regulates target gene expression.

Under extended exposure to hypoxic conditions, HIF-1*α* is expressed as long as a balance between O_2_ supply and usage in tissues cannot be reached [36, 37]. When oxygen tension is sufficient,* de novo* synthesised cytoplasmic HIF-1*α* is hydroxylated by a family of prolyl hydroxylase enzymes (PHDs) on proline 402 and 564 residues located within ODDD (O_2_-dependent degradation domain) [41, 42]. The hydroxylation of the abovementioned prolines is the key mechanism of negative regulation of HIF-1*α* activity and results in the binding of the von Hippel-Lindau (VHL) E3 ligase complex, which ubiquitinates HIF-1*α*, targeting it to proteasomal degradation [43–45]. The second major mechanism that modulates HIF-1*α* activity is the hydroxylation of asparagine residue 803 (Asn803) in the C-TAD domain. In this case, asparagine is hydroxylated under normoxic conditions by factor inhibiting HIF-1 (FIH-1), which prevents the interaction of HIF-1*α* within CBP/p300 (CREB-binding protein/E1A binding protein p300) [46–49]. Hydroxylases (PHDs and FIH-1) are strictly Fe(II)- and 2-oxoglutarate-dependent dioxygenases that are activated only in the presence of molecular oxygen. Under hypoxic conditions, substrates and coactivators of hydroxylation such as O_2_, Fe(II), and 2-oxoglutarate become limited, which leads to the attenuation of HIF-1*α* hydroxylation [50, 51]. HIF-1*α* accumulates in the cytosol and is subsequently translocated into the nucleus where it dimerises with the HIF-1*β* subunit. The HIF-1*α*/*β* dimer binds to HREs that are located within O_2_-regulated genes (Figure 2) [34, 35]. HIF-1 target gene members include a stress response gene family that mediates the adaptation of cells/tissues to chronic or acute hypoxia; this family includes glucose transporters, glycolytic enzymes, and angiogenic and haematopoietic growth factors [52].

### 2.3. HIF-1 Activity and Target Genes

Recently, HIF-1 has been shown to regulate more than 2% of genes in vascular endothelial cells either directly or indirectly [53]. The modulation of cell responses is followed by the transcriptional activation of target genes by the HIF-1*α*/*β* dimer (Figure 1(b)) [54, 55]. For example, hypoxia upregulates the expression of erythropoietin (EPO), which is required for red blood cell production. The generation of new erythrocytes increases the delivery of oxygen to tissues to reach O_2_ homeostasis [56]. Additionally, low oxygen levels influence glucose metabolism in cells. Under hypoxic conditions, cells generate only 2 ATP molecules by oxygen-independent glycolysis instead of 38 ATP molecules by the oxygen-dependent tricarboxylic acid cycle (TCA) under normoxic conditions [57, 58]. To maintain the energetic balance under hypoxic conditions, cells increase their ability to produce ATP by increasing glucose uptake via the enhancement of glycolytic enzyme and transporter expression [59–61]. Furthermore, cell proliferation and survival may be enhanced under hypoxic conditions. Factors such as IGF-2 (insulin growth factor-2) and TGF*α* (transforming growth factor) are upregulated through HIF-1 activity [62, 63]. Additionally, myoblasts cultured under hypoxic conditions show increased proliferation compared to cells maintained under normoxic conditions. Myogenic gene expression analysis revealed that the genes* MyoD* and* Myf5* (both involved in myogenic cell proliferation and differentiation) were upregulated in hypoxic cells, whereas no significant influence of myogenic gene expression was observed in normoxic cells [64].

## 3. Hypoxia-Induced Angiogenesis

### 3.1. Physiological Vasculogenesis and Angiogenesis under Hypoxic Conditions

Vasculogenesis is a characteristic embryonic process that involves* de novo* blood vessel formation and that leads to establishing a primary vascular plexus. O_2_ tension plays a crucial role in organogenesis and vasculogenesis during embryonic development [1]. During the first stage of embryogenesis, before the circulatory system develops, the oxygen tension is relatively low and does not exceed 3% [65, 66]. The developing embryo requires an increase in the oxygen level, which leads to the formation of primary vessels from angioblasts. Because the uterine environment is hypoxic it is thereby obvious that hypoxia may be the primary stimulus of vessel formation [3, 4]. Hypoxia also stimulates EC (endothelial cell) behaviour. Under* in vitro* conditions, HIF-1*α* promotes the arterial differentiation of endothelial progenitor cells (EPCs) over venous differentiation by regulating the expression of genes that inhibit the venous specification factor Coup-TFII (Hey2 and delta-like ligand 4 (DII4)) [67]. In contrast, HIF-1*β* enhances the differentiation of hemangioblasts from mesodermal progenitors [68]. Mouse model studies have demonstrated the participation of both HIF-1 dimer subunits in vasculogenesis [69, 70]. For instance, HIF-1*α*- or HIF-1*β* (ARNT) deficient mouse embryos showed aberrant placental architecture with fewer foetal blood vessels [71–73]. Additionally, a lack of the HIF-1*β* gene results in defective vascular development in the yolk sac, branchial arches, cranium, and somites [74, 75]. These defects are lethal for embryos at day 10.5. HIF-1 subunit deficiencies markedly decrease VEGF mRNA and protein expression, leading to defects in blood vessel formation and neural fold termination [70, 74, 75].

Current studies have indicated that hypoxia and HIF-1 expression in adult organisms may contribute to angiogenesis in the following ways: by transcriptionally activating several angiogenic genes and their receptors (*VEGF*,* PlGF*,* PDGFB*,* ANGPT1*, and* ANGPT2*) [76, 77]; by regulating proangiogenic chemokines and receptors (*SDF-1α*, stromal cell derived factor 1*α*, and* S1P*, sphingosine-1-phosphate, and receptors* CXCR4*, C-X-C chemokine receptor type 4, and* S1PRs*, sphingosine-1-phosphate receptors), thus facilitating the recruitment of endothelial progenitor cells to the site of hypoxia [78]; and by enhancing EC proliferation and division (regulating genes involved in the cell cycle and DNA replication) [53]. Summarising these findings, we conclude that HIF-1 can orchestrate the process of angiogenesis.

HIF-1 participates in every step of angiogenesis. Cross activity between HIF-1 and proangiogenic factors is a basic relationship during capillary formation under hypoxia. Every step of the vessel formation cascade is supported by HIF-1. Notably, vascular endothelial growth factor isoforms (VEGF-A, VEGF-B, VEGF-C, and VEGF-D) are the primary factors that participate in angiogenesis [79], and angiogenesis initiated by hypoxia and by HIF-1 is often VEGF-dependent primarily because HIF-1 is a master stimulator of vascular endothelial growth factor. The sprouting of new vessels is a complex process that involves a wide range of proangiogenic factors and their receptors. Under hypoxic conditions, HIF-1 accumulation upregulates the principal proangiogenic factor VEGF directly [80, 81]. VEGF activity induces the expression of Flt-1 (fms-related tyrosine kinase) and KDR (kinase insert domain receptor) receptors [82, 83]. As long as the oxygen balance is disrupted, VEGF will bind its receptors and stimulate capillary outgrowth. Additionally, VEGF is known as a factor that enhances the expression of other proangiogenic factors such as PlGF and FGF. We can assume that when the blood flow is sufficient to supply oxygen to cells and tissues, HIF-1*α* will be degraded, thereby inhibiting* VEGF* gene and protein expression and stopping the entire cascade of proangiogenic factors. In conclusion, HIF-1 may regulate the expression of proangiogenic factors either directly (by binding to HREs) or indirectly (cascade effect) [84]. Aside from VEGFs, other factors participate in angiogenesis, including placental growth factor (PlGF), platelet-derived growth factor (PDGF), angiopoietins 1 and 2 (ANGPT1 and ANGPT2), and metalloproteinases (MMPs). Receptors such as Flt-1, KDR, Tie 1, and Tie 2 that transduce these signals and thereby maintain the cascade of new vessel formation are also important. Analyses of proangiogenic genes revealed the presence of HREs within the promoters of some genes [85, 86].

As was mentioned above, angiogenesis is a multistep process [87]. During the first step, hypoxia and HIF-1 stimulate VEGF and their receptors directly, and the cascade of new vessel creation begins. Second, the extracellular matrix must be degraded by metalloproteinases to allow the migrating endothelial cells to form tubes. Metalloproteinase 2 (MMP-2) expression has been shown to be enhanced by HIF-1*α* [88]. Next, integrins *αβ* (induced by HIF) [89] stimulate endothelial cell proliferation and adhesion, and HIF-1 controls EC behaviour. The Manalo group examined the influence of hypoxia and HIF-1 on the endothelial cell transcriptome. Microarray analysis demonstrated the upregulation of several genes that are responsible for the cell cycle and DNA replication. Additionally, it was proven that ECs cultured under low density and hypoxic conditions exhibit increased proliferation. In conclusion, HIF-1 can stimulate cell proliferation and division under hypoxia-induced angiogenesis by transcriptionally regulating the genes involved in the basic biological functions of cells [53]. Finally, the last step of angiogenesis is vessel maturation, which involves the recruitment of vascular supporting cells (pericytes and smooth muscle cells) and the formation of the basement membrane. In this case, HIF-2*α* is a primary enhancer of genes that determine blood vessel stabilisation. For example, fibronectin is a component of the basement membrane, and HIF-2*α* is involved in the upregulation of this gene [90]. Considering these findings, we can assume that HIF-1 regulates almost every step of capillary formation.

Increasing knowledge regarding the influence of HIF-1 on angiogenesis has been applied to* in vitro* studies that attempt to develop treatments for ischaemic diseases. For example, the adenoviral transfer of HIF-1*α* and HIF-2*α* into rabbit ischaemic limbs resulted in an increase in blood flow followed by induction of vessel sprouting. Additionally, these capillaries were highly enlarged compared to those of rabbits transduced with* VEGF-A*. As another consequence of* VEGF* gene transfer, the newly formed vessels were leaky, and the area of transfer was surrounded by oedema compared to animals treated with AdHIF-1*α* and AdHIF-2*α* [91]. In turn,* in vivo* studies demonstrated that the deletion of HIF-2*α* in murine ECs caused VEGF-induced acute vessel permeability. Furthermore, immortalised HIF-2*α*-deficient ECs exhibited decreased adhesion to extracellular matrix proteins and diminished expression of fibronectin, integrins, endothelin B receptor, angiopoietin-2, and delta-like ligand 4 (Dll4) [90]. Artificial hypoxia induced by CoCl_2_ (0.5 mM) stimulates angiogenic responses in SCAPs (stem cells from apical papilla) cocultured with HUVECs (human umbilical vein endothelial cells). Hypoxic conditions induced upregulated HIF-1*α* and VEGF expression in HUVECs, whereas* ephrin-B2* gene was enhanced in SCAPs. Notably, this gene plays a central role in heart morphogenesis and angiogenesis by regulating cell adhesion and cell migration. Additionally, synergistic effects between HIF-1, VEGF, and ephrin-B2 led to an increase in the endothelial tubule number, vessel length, and branching points [92].

In conclusion, hypoxia-induced angiogenesis is a complex process that has been tested in both* in vitro* and* in vivo* studies. Moreover, the application of HIF-1 therapy in phase I clinical studies of critical limb ischaemia resulted in complete recovery of the ischaemic region [93]. Increased knowledge of the role of HIF-1 in angiogenesis may provide promising treatment methods for ischaemic diseases as partly described in Section 4.1 of this paper.

### 3.2. The Effect of Hypoxia on Pathophysiological Angiogenesis and the Role of HIF-1 in Tumour Angiogenesis

Low oxygen tension has been linked with many pathophysiological disorders and human diseases. Hypoxia is a component of tumour development and metastasis, while angiogenesis is principal for tumour growth and progression [94, 95]. Current studies have suggested that in terms of malignant transformation the activation of HIF-dependent angiogenesis in cancer occurs in two basic ways: by hypoxic conditions prevailing in the tumour cell mass or by genetic alterations caused by tumour transformation, genetic disorders, or molecular interactions that stimulate HIF activity, irrespective of oxygen tension. Undeniably, HIF plays a critical role in stimulating angiogenesis. However, notably, proangiogenic activity is directly linked with HIF-dependent VEGF activation, which results in an “angiogenic switch” in growing tumour masses [96].

Hypoxia is best characterised as an HIF activator. When intensively proliferating cells form a solid tumour, the balance between oxygen supply and demand is impaired; therefore, hypoxic environments (intratumoural hypoxia) prevail in growing cell masses. This prevalence is the reason why newly activated HIF-1*α* is not ubiquitinated and targeted to proteasomal degradation. Accumulating HIF-1 upregulates the expression of a number of proangiogenic genes including* VEGF* and their receptors* Flt-1*,* Flk-1*,* Ang-1*,* Ang-2*, and* Tie-2* receptor, and all of these genes are essential for sprouting new vessels. Among these factors, VEGF is considered a primary effector of tumour angiogenesis. Moreover, the expression of VEGF can enhance the expression of other proangiogenic factors and their receptors; thus, vessel outgrowth is stimulated by multiple factors [95, 97]. This phenomenon, which is called “angiogenic switching,” allows tumour cells to induce angiogenesis, thereby stimulating tumour progression by supplying oxygen and nutrients through the newly created capillaries [96].

The above-presented data suggest that hypoxia directly enhances angiogenesis by promoting VEGF expression. In contrast, HIF-1-dependent angiogenesis can be activated by factors other than hypoxia. HIF-1*α* accumulation does not always occur under low O_2_ tension. Thus, HIF-1 can also be regulated by oxygen-independent mechanisms. The malignant transformation of cells may cause a whole range of genetic alterations that block the ubiquitination and proteasomal degradation of HIF-1*α* [84, 98]. Carcinogenesis is linked with aberrations in tumour suppressor genes also known as antioncogene genes. The protein products of these genes may control cell division, the cell cycle, or apoptosis. When cells exhibit DNA damage, these proteins are responsible for repressing the cell cycle or cell division or for promoting apoptosis. The most important tumour suppressors are pRb, p53, p21, and PTEN. Some evidence has indicated that altered antioncogenes can enhance angiogenesis via HIF-dependent VEGF stimulation. For instance, deleting the p53 tumour suppressor gene in a human cancer cell line promotes the neovascularisation and growth of tumours in mice. This vascularisation was followed by increased HIF-1*α* levels and augmented HIF-1-dependent transcriptional activation of vascular endothelial growth factor (VEGF) [99]. Additionally, mutation in the tumour suppressor gene PTEN led to hypoxia-independent HIF-1*α* accumulation and to activated HIF-1-mediated proangiogenic gene expression [100]. In breast cancer, HER2 (receptor tyrosine kinase) signalling induces HIF-1*α* protein synthesis rather than inhibiting its degradation, thus manifesting a novel mechanism of HIF-1-dependent VEGF expression regulation [101]. These findings suggest that not only the hypoxic environment can lead to angiogenesis in the case of malignant transformation. Additionally, the genetic alteration of single gene may be a cause of hypoxia-independent stimulation of HIF. Some evidence has indicated that genetic diseases are involved in the activation of HIF and thereby the stimulation of angiogenesis. von Hippel-Lindau is a genetic disease that predisposes individuals to benign and malignant tumours [102]. This condition is defined by a mutation in the* VHL* gene; thus, pVHL is not translated. This protein is a ligase that ubiquitinates HIF-1*α* and causes its degradation by proteasomes; thus, maintaining the balance of HIF-1 under normoxia is crucial. A loss of pVHL allows HIF-1*α* to dimerise with HIF-1*β* and to activate the transcription of a number of proangiogenic genes, including* VEGF*. Patients suffering from this disease have frequent malignancies in the central nervous system and/or retinal hemangioblastomas or clear cell renal carcinomas [103–106]. However despite homology between HIF-1*α* and HIF-2*α* there are evidences that in VHL-defective renal cell carcinoma HIF isoforms exhibit different or opposite effect on gene expression and proliferation of tumour cells [107]. Genetic predisposition is not the only factor that can stimulate oxygen-independent HIF angiogenesis involving* VEGF* gene activation. Cases where some other molecules can stimulate HIF-dependent angiogenesis have been demonstrated. As an example, we can recall a phenomenon based on the feedback loop of HIF-1 and a product of the anaerobic metabolic pathway. The abovementioned mechanism that activates HIF-1-dependent angiogenesis in cancer cells is the phenomenon called the Warburg effect. Because anaerobic conditions prevail in tumour cell masses, cancer cells produce energy primarily by glycolysis. As a side effect, cells also produce high levels of lactates and pyruvates. These molecules have been reported to increase HIF-1*α* accumulation and to regulate hypoxia-inducible gene expression, thus promoting* VEGF* transcription and translation and resulting in tumour angiogenesis [108].

Strong interactions between HIF-1*α* and VEGF lead to the rapid activation of the vessel formation cascade; however, these vessels exhibit several dysfunctions. The process of intratumoural vessel formation is a consequence of imbalanced activity of angiogenic activators and inhibitors. These vessels are not fully functional and exhibit structural abnormalities. A loss of stabilisation with pericytes and smooth muscle cells and a lack of an arterial and venous phenotype lead to leakage, poor blood flow, and perfusion [109, 110]. However, these vessels are able to deliver nutrients and oxygen to growing tumour cell masses. Thus, we can also conclude that tumours with vasculature are more sensitive to various therapies. Due to vascularisation, chemotherapeutics and inhibitors may be better distributed across tumour cells, thus leading to the inhibition of malignant cell growth.

## 4. Hypoxia as a Potential Therapeutic Tool

**Figure 3:**
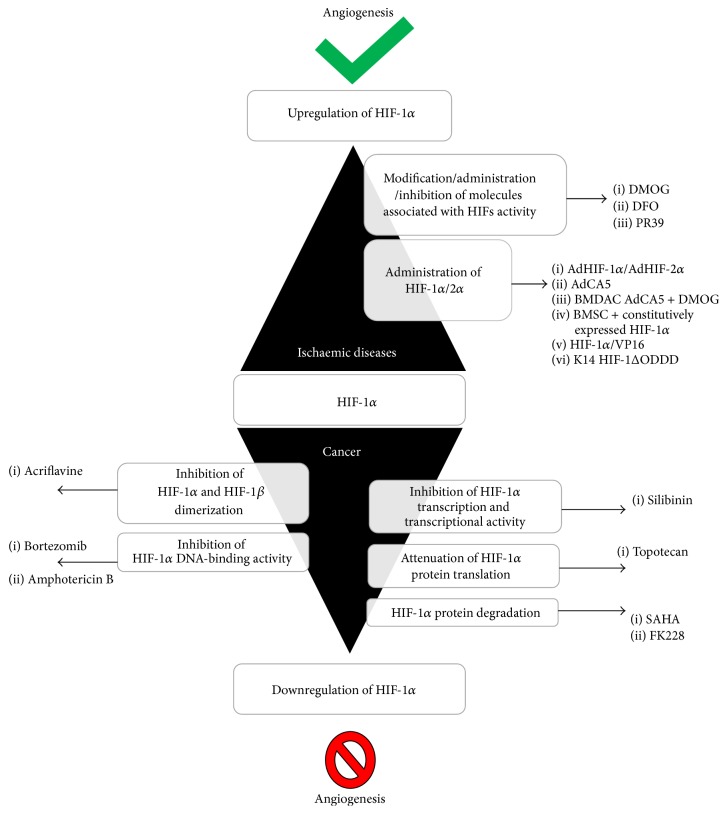
*Different strategies in HIF-1α therapies*. Depending on the type of disease and the expected therapeutic effect, different approaches are used in HIF-1 therapies: HIF-1 upregulation (ischaemia) to induce angiogenesis or HIF-1 inhibition to attenuate angiogenesis (cancer). DMOG: dimethyloxalylglycine; DFO: 2-OG analogue (N-oxalylglycine); PR39: macrophage-derived peptide; AdHIF-1*α* and AdHIF-2*α*: adenoviral vector; AdCA5: adenovirus encoding a constitutively active form of the HIF-1*α*; BMDAC: bone marrow-derived angiogenic cells; HIF-1*α*/VP16: DNA-binding and dimerisation domains of HIF-1*α* fused with the transactivation domain of herpes simplex virus; K14-HIF-1ΔODD: vector encoding O_2_-independent HIF-1*α* under the keratin 14 promoter. Silibinin: nontoxic flavonoid; topotecan: chemotherapeutic agent that is a topoisomerase inhibitor; SAHA and FK228: histone deacetylase inhibitors; acriflavine, bortezomib; amphotericin B: anti-HIF drugs.

Hypoxia-inducible factor-1 has been considered a potential therapeutic target in many diseases. Predominantly, HIF-1 therapies focus on diseases common to developing countries such as ischaemic disorders (including cardiovascular disease and limb ischaemia) and cancer. Nonetheless, HIF-1 is also a potential therapeutic agent for treating endometriosis and blindness. Depending on the type of disease and the expected therapeutic effect, HIF-1 therapies have different approaches. The function of HIF-1 in therapies can be classified into two different strategies: HIF-1 upregulation (ischaemia) and HIF-1 inhibition (cancer and endometriosis) (Figure 3).

### 4.1. Activation of HIF-1-Dependent Angiogenesis in Ischaemic Diseases

The neovascularisation of ischaemic regions is a fundamental assumption of therapeutic angiogenesis. The induction of capillary outgrowth for therapeutic purposes is stimulated by the administration of angiogenic growth factors or sequences that encode these proteins. Thus far, multiple angiogenic factors such as VEGF, PlGF, FGF, and PDGF have been applied in* in vitro* studies and in preclinical and clinical trials [111–114]. However, therapies using only one proangiogenic agent to initiate angiogenesis were shown to be insufficient; thus, these therapies may require supplementation with other factors that can stabilise new capillaries. Therefore, hypoxia-induced angiogenesis may be a successful strategy [115]. HIF-1 regulates cell-type specific proangiogenic factors and cytokines either directly or indirectly. Promoting HIF-1 activity is essential in ischaemic diseases. Thus far, HIF-1 therapies have been based on two approaches: the administration of HIF-1*α*/2*α* or the induction of HIF-1 expression by the modification/administration/inhibition of molecules associated with HIF activity.

Basic strategies of therapeutic angiogenesis involve the administration of proangiogenic factors using vectors, combined therapies with stem cells or fusion/recombinant proteins. Hypoxia-inducible gene transfer led to the enhancement of angiogenesis in both myocardium and ischaemic skeletal muscles. Additionally, AdHIF-1*α* and AdHIF-2*α* injection did not induce tissue oedema in contrast to regions treated with AdVEGF. In conclusion, HIF application in this case resulted in the formation of stable and mature vessels compared to VEGF treatment, where the newly formed vessels were leaky [91]. To prevent oxygen-dependent degradation of HIF-1*α*, attempts to modify this subunit were made. An alternative approach of applying AdCA5 adenovirus encoding a constitutively active form of the HIF-1*α* subunit due to a deletion and a point mutation in the region responsible for O_2_-dependent degradation was proposed. In a model of endovascular limb ischaemia, intramuscular injection of AdCA5 improved the recovery of blood flow by stimulating both angiogenesis and arteriogenesis [116]. Furthermore, therapy with AdCA5 induced the upregulation of several proangiogenic genes/proteins that are targets for therapeutic angiogenesis in hindlimb and cardiac ischaemia models, including FGF-2, hepatocyte growth factor, MCP-1, PDGF-B, PlGF, SDF-1, and VEGF. Moreover, AdCA5 injection into mouse eyes enhanced neovascularisation in multiple capillary beds, including those not responsive to VEGF alone. Due to the upregulation of* PlGF* and* VEGF* expression after AdCA5 treatment, both genes acted synergistically, which led to neovascularisation of the retina [117]. Combined therapy using AdCA5 gene therapy and prolyl-4-hydroxylase inhibitor dimethyloxalylglycine- (DMOG-) treated BMDACs (bone marrow-derived angiogenic cells) acted synergistically to increase the recovery of blood flow after femoral artery ligation, thereby preventing tissue necrosis. This synergistic effect is due to AdCA5 enhancement of BMDAC homing, whereas DMOG treatment increases the retention of grafted cells in the ischaemic tissues. Another combined therapy with modified stem cells was applied in cerebral ischaemia. Rat bone marrow-derived mesenchymal stem cells (BMSCs) were infected with adenoviral particles containing constitutively expressed HIF-1*α* due to mutations in proline 564 and asparagine 803 sites. Genetically modified cells were transplanted in a rat middle cerebral artery occlusion model (MCAO). At 7 days after intervention, improved motor function, reduced cerebral infarction, and increased VEGF protein expression that led to revascularisation were observed [118]. The stabilisation and administration of HIF-1*α* may be achieved by the construction of a fusion protein. For instance, DNA-binding and dimerisation domains of HIF-1*α* were fused with the transactivation domain of herpes simplex virus (VP16). A plasmid vector encoding this structure was used in a rabbit hindlimb ischaemia model. The administration of HIF-1*α*/VP16 resulted in the improvement of blood flow in the ischaemic region as determined by an increase in the number of blood vessels [119]. Additionally, using promoters specific for particular cells or tissues, angiogenesis could have occurred in the designated site. The construction of a vector encoding O_2_-independent HIF-1*α* under the keratin 14 promoter (K14-HIF-1ΔODD) resulted in the upregulation of HIF-1*α* in a skin. The activation of HIF-1*α* increased skin capillary density. Moreover, the new vessels were not leaky, and enhanced skin vascularity was not associated with oedema compared to the capillaries that formed after K14-VEGF therapy [120].

Another approach leading to HIF-1-dependent angiogenesis is associated with the modification/administration of molecules that regulate HIF-1*α* activity. The stabilisation of HIF-1*α* under normoxic conditions is focused on the inhibition of prolyl hydroxylase activity. As mentioned above, PHDs require O_2_, Fe(II), and 2-oxoglutarate (2-OG) for their enzymatic activity [50, 51]. The delivery of small molecules such as an iron chelator (DFO) or 2-OG analogue (N-oxalylglycine) may inhibit the enzyme activity [121, 122]. For instance, HIF-1 can be enhanced by the suppression of prolyl hydroxylase activity using dimethyloxalylglycine (DMOG). The systemic administration of DMOG in a mouse model of hindlimb ischaemia results in the elevation of HIF-1*α* protein expression, which leads to neovascularisation in the infarcted region [121]. Then, capillary growth is followed by HIF-induced VEGF and Flk-1 upregulation. A novel strategy involves the preconditioning of rat bone marrow-derived mesenchymal stem cells (BMSCs) using DMOG to enhance their survival and therapeutic efficacy after transplantation into infarcted rat hearts. After DMOG treatment, these cells exhibited enhanced expression of survival and proangiogenic factors such as HIF-1*α*, vascular endothelial growth factor, and glucose transporter 1. Thus, the transplantation of DMOG-treated BMSCs reduced heart infarction size and promoted angiogenesis in the ischaemic region [123]. An alternative to the use of PHD inhibitor molecules is the administration of a molecule that stabilises HIF-1*α* under normoxic conditions. PR39, which is a macrophage-derived peptide, achieved this effect by inhibiting HIF-1*α* degradation through the ubiquitin-proteasome system. The introduction of the PR39 peptide resulted in a significant increase in HIF-1*α* protein levels irrespective of ischaemia or hypoxia. Therefore, angiogenesis can be induced in the postinfarcted myocardium of transgenic mice [124].

### 4.2. Inhibition of HIF-1-Dependent Angiogenesis in Cancer Therapies

Cancer therapies are based on the targeted inhibition of proangiogenic factors. Current treatments are focused on the inhibition of VEGF activity. Because of studies regarding VEGF antiangiogenic therapy, patients were treated with bevacizumab (VEGF inhibitor) or sunitinib (VEGFR2 inhibitor) [125]. Since the discovery that the HIF-1 pathway regulates the activation of many proangiogenic factors in tumours and may promote metastasis, the HIF-1*α* subunit has been considered an attractive target for new cancer therapeutics. The inhibition of HIF-1-dependent angiogenesis involves the regulation of HIF-1*α* activity by molecules that modulate HIF-1*α* transcription and transcriptional activity, HIF-1*α* and HIF-1*β* dimerisation, HIF-1*α* protein translation, HIF-1*α* DNA binding, and HIF-1*α* protein degradation [24, 126].

Thus far, the Food and Drug Administration has approved several anti-HIF drugs. For instance, bortezomib and amphotericin B functionally inhibit HIF-1*α*, thereby preventing p300 recruitment by enhancing the interaction between FIH-1 and the HIF-1*α* C-terminal transactivation domain (C-TAD) [127, 128]. In contrast, silibinin is a nontoxic flavonoid that is able to inhibit hypoxia-dependent HIF-1*α* accumulation and to inhibit HIF-1 transcriptional activity in HeLa and hepatoma cells [129]. Silibinin is also a potent inhibitor of cell proliferation. Two other drugs, SAHA and FK228, are histone deacetylase inhibitors that were found to promote HIF-1*α* degradation by upregulating p53 and VHL. These drugs have been successfully used in the United States. HIF-1 activity might also be attenuated by previously used chemotherapeutics such as anthracycline and doxorubicin, which act by inhibiting HIF-1*α* DNA-binding activity, and acriflavine, which prevents HIF subunit dimerisation [130, 131].

Clinical trials are presently focused on other drugs that may attenuate HIF-1-dependent angiogenesis. Currently, flavopiridol (Alvocidib) is in phase III clinical trials. This synthetic flavonoid, which is derived from an alkaloid Indian plant, was found to be a potent inhibitor of the transcription of HIF-1*α* and of many other genes involved in cell cycle arrest [132, 133]. Other clinical studies (phases I and II) are being conducted with many other drugs that involve HIF-1 degradation, downregulation, or inactivation.
